# Pollination Drop Proteome and Reproductive Organ Transcriptome Comparison in *Gnetum* Reveals Entomophilous Adaptation

**DOI:** 10.3390/genes10100800

**Published:** 2019-10-12

**Authors:** Chen Hou, Richard M. K. Saunders, Nan Deng, Tao Wan, Yingjuan Su

**Affiliations:** 1 School of Life Sciences, Sun Yat-Sen University, Xingangxi Road No. 135, Guangzhou 510275, China; houchen1986@gmail.com; 2 Division of Ecology & Biodiversity, School of Biological Sciences, The University of Hong Kong, Pokfulam Road, Hong Kong, China; saunders@hku.hk; 3 Institute of Ecology, Hunan Academy of Forestry, Shaoshannan Road, No. 6581, Changsha 410004, China; idengnan@sina.com; 4 Hunan Cili Forest Ecosystem State Research Station, Cili 427200, China; 5 Key Laboratory of Southern Subtropical Plant Diversity, Fairy Lake Botanical Garden, Shenzhen & Chinese Academy of Science, Liantangxianhu Road, No. 160, Shenzhen 518004, China; wantao1983@gmail.com; 6 Sino-Africa Joint Research Centre, Chinese Academy of Science, Moshan, Wuhan 430074, China

**Keywords:** insect pollination, label-free quantitative sequencing, pollination drops, proteome, transcriptome, Gnetales

## Abstract

*Gnetum* possesses morphologically bisexual but functionally unisexual reproductive structures that exude sugary pollination drops to attract insects. Previous studies have revealed that the arborescent species (*G. gnemon* L.) and the lianoid species (*G. luofuense* C.Y.Cheng) possess different pollination syndromes. This study compared the proteome in the pollination drops of these two species using label-free quantitative techniques. The transcriptomes of fertile reproductive units (FRUs) and sterile reproductive units (SRUs) for each species were furthermore compared using Illumina Hiseq sequencing, and integrated proteomic and transcriptomic analyses were subsequently performed. Our results show that the differentially expressed proteins between FRUs and SRUs were involved in carbohydrate metabolism, the biosynthesis of amino acids and ovule defense. In addition, the differentially expressed genes between the FRUs and SRUs (e.g., MADS-box genes) were engaged in reproductive development and the formation of pollination drops. The integrated protein-transcript analyses revealed that FRUs and their exudates were relatively conservative while the SRUs and their exudates were more diverse, probably functioning as pollinator attractants. The evolution of reproductive organs appears to be synchronized with changes in the pollination drop proteome of *Gnetum*, suggesting that insect-pollinated adaptations are not restricted to angiosperms but also occur in gymnosperms.

## 1. Introduction

**Figure 1:**
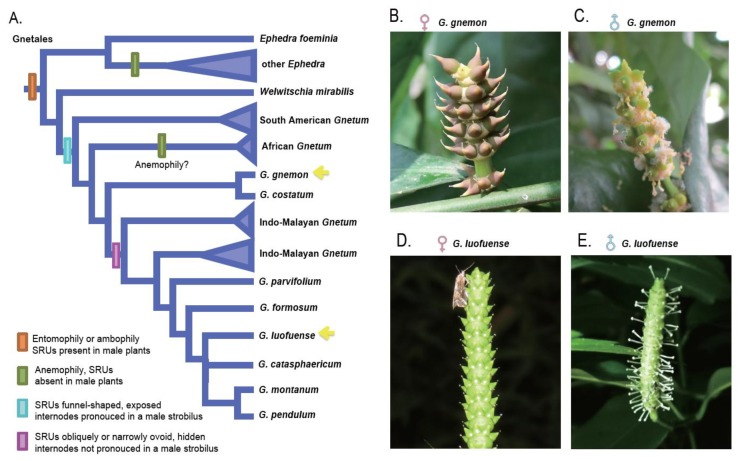
Reproductive evolution and morphology of *Gnetum*. (**A**) A schematic phylogeny illustrating the pollination biology and inferred reproductive evolution of the Gnetales (modified from Jörgensen and Rydin, 2015). Yellow arrows denote the phylogenetic placement of the two *Gnetum* species investigated in the present study. (**B**–**E**) Reproductive structures and pollination drops of *G. gnemon* and *G. luofuense*. (**B**) A female strobilus of *G. gnemon*. (**C**) A male strobilus of *G. gnemon* with sterile reproductive units (SRUs) producing pollination drops. (**D**) A female strobilus of *G. luofuense* with a moth sucking pollination drops from a fertile reproductive units (FRU). (**E**) A male strobilus of *G. luofuense* with SRUs producing pollination drops. Photographs by C. Hou.

Animal pollination (and entomophily in particular) has a profound impact on species diversity and the geographical distribution of extant seed plants [1,2,3]. In marked contrast to angiosperms, however, entomophily has rarely been reported in gymnosperms, and is restricted to cycads [4,5,6] and Gnetales [7,8]. The reports of insect pollination in Gnetales (comprising three genera, *Ephedra* L., *Welwitschia* Hook.f. and *Gnetum* L.) include *E. foeminea* Forssk. [9,10,11], *W. mirabilis* Hook.f. [12] and several *Gnetum* species [13,14,15,16,17] (Figure 1). In contrast, the majority of *Ephedra* species are wind pollinated [9,10,18] and African *Gnetum* species are likely to be predominantly anemophilous [19,20] or with wind pollination as a supplement to entomophily (e.g., *G. parvifolium*(Warb.) W.C.Cheng [21]). Insect pollination has been inferred to be the plesiomorphic condition within the Gnetales, with wind pollination derived [9,11,19].

Pollination drops are essential in gymnosperm reproduction, providing a site for pollen attachment and a medium for germination [22,23,24,25,26]. Gymnosperm pollination drops are composed of carbohydrates, minerals, lipids, amino acids and proteins [25,26,27,28]. Among these chemicals, sugars such as sucrose are the main reward for insect pollinators, and are therefore comparable to floral nectar in angiosperms [25,26,27,29]. The proteins in the pollination drops of gymnosperms are furthermore involved in varied physiological and ecological processes, such as pollen tube growth [30,31], carbohydrate metabolism [30] and ovule protection against fungi and pathogens [31,32,33]. These proteins, found in the apoplast, are secreted by the nucellus and are referred to as secretome proteins. In contrast, the proteins that are not detected in the apoplast are derived from the breakdown of the nucellus during the formation of a pollen chamber, and are known as degradome proteins [28,34,35]. Degradome proteins in the pollination drops of gymnosperms are probably associated with degenerated megaspores [36] and the nucellus degeneration mediated by programmed cell death (PCD) [37]. For an example, acid phosphatase activity was found in the nucellus of the female reproductive units of an *Ephedra* species [38], and degradome proteins have been shown to be more abundant than secretome proteins in the pollination drops of several *Ephedra* species [34].

Pollination drops in the Gnetales have a typical function, with fertile reproductive units (FRUs) and sterile reproductive units (SRUs) producing sugar-rich drops as a reward for insect pollinators [7,8,9,11,19]. A recent study [35] has shown that protein numbers in the pollination drops of FRUs (henceforth female drops) in *G. gnemon* L. and *W. mirabilis* (17 and one, respectively) are considerably fewer than in those of SRUs (male drops, 25 and 138 in the two species, respectively). The reasons why the protein numbers in female and male drops differ so markedly and their association with insect pollination have yet to be convincingly explained. This might be associated with the fact that the total sugar concentration (TSC) in the male drops of *G. gnemon* was significantly lower than the female drops, although the relative proportion of different sugar types was identical between the female and male drops [27]. In addition, many degradome proteins were reported in the female drops of seven *Ephedra* species, as well as male drops of *G. gnemon* and *W. mirabilis* [34,35]. The degradome proteins are the consequence of cell apoptosis during the formation of the pollen chamber, as reported in *Ephedra* [34,38] and *Gnetum* [39]. A deeper investigation of the pollination drop proteome would help infer reproductive evolution and entomophilous adaptation in the Gnetales. 

The pantropical genus *Gnetum* comprises 30–40 species of trees, shrubs and lianas [40,41,42]. Molecular phylogenies have identified a South American clade, sister to a clade comprising African and Asian species [41,43,44] (Figure 1A). Within the Asian lineage, two arborescent species, *G. gnemon* and *G. costatum* K.Schum., form a clade (*G.* subsect. *Eugnemones* Markgr.) that is sister to all the lianoid species (*G.* sect. *Cylindrostachys* Markgr.: Hou et al., 2015). A study of the pollination biology of the arborescent species *G. gnemon* and the lianoid species *G. cuspidatum* Blume, revealed that the sugar-rich female drops of *G. cuspidatum* attracted diverse insects, especially lauxaniid flies, whereas the relatively sugar-weak female drops of *G. gnemon* were exclusively visited by pyralid and geometrid moths [13,14]. Kato et al. proposed that the unspecialized attraction to insects observed in *G. cuspidatum* might represent an ancestral condition shared by the Gnetales and early angiosperms [14]. This unspecialized attraction has been corroborated by recent investigations of the Asian lianoid species *G. parvifolium* [21] and *G. luofuense* C.Y.Cheng [45]. In contrast, however, *G. gnemon* was proposed to have undergone secondary evolution as the consequence of its adaptation to long-tongued nocturnal moths, associated with moth-angiosperm co-evolution during the late Cretaceous [14].

*Gnetum* possesses morphologically bisexual but functionally unisexual reproductive structures [8,16,19,46,47]. Each female/male strobilus comprises multiple whorls of involucral collars (Figure 1B).The involucral collar in a female strobilus has multiple FRUs in a whorl, while in the male strobilus, a single whorl of SRUs was subtended by several whorls of microsporangiophores [8,46,48,49,50,51,52]. The bisexual structure in *Gnetum* facilitates crossing between the different individuals via insect pollination: The wind-pollinated African species, in contrast, lack such a structure [51,53,54]. The SRUs of Asian arborescent species are exposed and funnel-shaped, whereas those of Asian lianoid species are hidden within an involucres and are obliquely, and/or narrowly ovoid [19,47]. The nucellus of the FRU in *Gnetum* is further more enclosed by three envelopes [55,56,57], whereas the nucellus of the SRU is enclosed by only two envelopes [19,58]. These striking differences in the gross morphology and anatomy of the FRU and SRU within *Gnetum* are presumably mediated by different genes. Previous studies have revealed that MADS-box genes manipulate the development of reproductive organs and sexual identity of *G. gnemon* and *G. parvifolium* [59,60,61,62]. Nevertheless, the differentially expressed genes between a FRU and a SRU and their potential associations with insect pollination have never been investigated.

This study hypothesized that SRUs and male drops of *Gnetum* exhibit a more pronounced entomophilous adaptation than FRUs and female drops, because the emergence of SRUs and male drops are solely responsible for insect attraction while FRUs and female drops are mainly involved in seed development. To test this hypothesis, a comprehensive survey was first conducted to compare the protein profiles in the female and male drops of *G. gnemon* and *G. luofuense* using label-free quantitative techniques. The transcripts of the FRUs and SRUs using Illumina sequencing techniques were assembled and compared, and the integrated protein-transcript analyses were performed to assess the selection imposed by insects on the reproductive organs and pollination drops of the two species. This is the first study to investigate the molecular mechanisms underlying entomophilous adaptation in gymnosperms. Since the SRUs and male drops of the Gnetales are unique in gymnosperms, the investigations of gnetalean reproductive organs and their pollination drops are expected to shed light on the mechanisms that drive the morphological and species diversity of extant gymnosperms.

## 2. Materials and Methods 

### 2.1. Pollination Drop Sampling

The pollination drops from a female and a male individual of *G. gnemon* (corresponding voucher specimens CH001 and CH002 deposited in SYS herbarium) were collected on 23–27 April, 2018 at Xishuangbanna Tropical Botanical Garden, Chinese Academy of Sciences, Yunnan, China. The pollination drop samples from a female and male individual of *G. luofuense* were obtained at Bamboo Garden, Sun Yat-sen University, Guangzhou, China on 8–10 May, 2018 (corresponding voucher specimensCH003 and CH004 deposited in SYS). The sampling was performed from 1930–2130 h during peak pollination times [14,21], although the pollination drops were sometimes additionally collected from 0830–1030h when the ambient humidity exceeded 90%. Our drop sampling strategy follows the methods applied in previous studies [30,31,33,34,35,63,64]. The female and male drops of *G. gnemon/G. luofuens* were separately collected using 10 μL micropipette tips. To generate the required sample volume for each species of *Gnetum*, nocturnal and daily collections were taken from 15–20 female strobili and 30–40 male strobili in two separated 1.5 mL Eppendorf tubes. 

### 2.2. Gel Electrophoresis

The pollination drop samples were initially examined using standard sodium dodecyl sulfate polyacrylamide gel electrophoresis (1D SDS-PAGE). For each pollination drop sample, 25 μL was incubated at 100 °C for 5 min and centrifuged at 14,000× *g* for 5 min. The samples were then mixed with 6 μL 5× MES SDS loading buffer (TransGen Biotech, Beijing, China) and a 5 μL protein ladder (ThermoFisher Scientific, Waltham, USA), and loaded onto a NuPAGE gel (Life Technologies, Shanghai, China). Gel electrophoresis was conducted at 14mA for 90 min. The gel was then stained using 0.1% G250 Coomassie Brilliant Blue and washed in10% acetic acid.

### 2.3. Trypsin Digestion and LC-MS/MS

The protein extraction, trypsin digestion and proteomic sequencing were performed by Biomarker Technologies Inc., Beijing, China. A total of 60 μg of each sample was mingled with 5 μL 1M DTT (DL-Dithiothreitol; Gen-View, Shanghai, China) and stored at 37 °C for 1 h. The solution was mixed with 20 μL 1M IAA (iodoacetamide; Vetec, Sigma-Aldrich, Saint Louis, MO, USA) and stored in the dark for 1 h. The prepared samples were rinsed twice in 100 μL UA solution (8M urea and 100 mMTris-HCl, pH8.0; Sigma-Aldrich) and three times in 100 μL NH_4_HCO_3_ (50 mM; Sigma-Aldrich). The cleaned samples were digested using sequencing-grade modified trypsin (Promega, Madison, WI, USA) in a volume ratio of 50:1 at 37 °C for 12–16 h.

The digested samples were separated chromatographically using an Ultimate 3000 system (Thermo Scientific, Waltham, MA, USA). The peptide samples were mixed with solution A (0.1% formic acid) on an in-house prepared C18 precolumn (3 μm; 100 μm × 20 mm) and then further separated by an in-house prepared C18 column (1.9 μm; 150 μm × 120 mm) in solution B (0.08% formic acid and 80% acetonitrile) at a flow rate of 600 nL min^−1^. The gradient comprised of the flowing settings: 8–12% solution B for 8 min; 12–23% for 40 min; 23–36% for 20 min; followed by an increase to 95% solution B for 1 min, holding for 9 min. The cumulative experimental duration was therefore 78 min. The LC-MS/MS analyses were performed using an Orbitrap Fusion mass spectrometer (Q-Exacitve HF, Thermo Scientific). The *m*/*z* range of the scanned spectra was set at 350–1500 Da at a resolution of 120,000, with an automatic gain control target value of 400,000. The spectra of the collision-induced dissociation were obtained using 32% normalization collision energy. The isolation window was set at 1.6 with the intensity threshold at 50,000. The quantification of the proteins was performed via the calculation and normalization of chromatographic peak intensity using MaxFLQ [65]. 

### 2.4. Proteomic Analyses and Bioinformatics

The raw proteomic data were analyzed using Mascot v.2.2 (Matrix Science, London, UK) embedded in Proteome Discoverv.2.0 (Thermo Scientific). All raw data were searched against the reference genome of *G. montanum* Markgr. (=*G. luofuense*) [66] with the following search parameters: The maximum number of missed cleavages was 2; the peptide mass tolerance was 15ppm; the fragment mass tolerance was 0.5Da; and there was a fixed modification of carbamidomethylation (C) and variable modification of oxidation (M) and protein N-term acetylation. The threshold of the false discovery rate (FDR) was set at 0.01 [67].

The protein functions and classification were determined using four databases: Swiss-Prot (a manually annotated, non-redundant protein sequence database, http://www.uniprot.org/); Nr (NCBI non-redundant protein sequences, ftp://ftp.ncbi.nih.gov/blast/db/); GO (Gene Ontology, http://www.geneontology.org/); and KEGG (Kyoto Encyclopedia of Genes and Genomes, http://www.genome.jp/kegg/). To define the differentially abundant proteins (DAPs), the abundance of proteins was compared with the fold-change (FC), with 1.5 as the threshold. The KEGG enrichment analyses of DAPs were performed using software ggplot2 v.1.0.1 [68], with corrected *p* values from the Fisher’s exact test <0.05.

### 2.5. Reproductive Organ Collection and RNA Sequencing

The material for RNA sequencing was obtained from the same female and male strobili of *G. gnemon* and *G. luofuense* used previously for the pollination drop sampling. For each species, the FRUs and SRUs were manually segregated from three randomly selected female and three male strobili. The FRUs and SRUs for each species were pooled, respectively, and the four transcriptomic samples were preserved in RNAlater (ThermoFisher Scientific, Shanghai, China) at −20 °C prior to RNA sequencing. It is noteworthy that various components of the female reproductive unites were not separated, as performed in a previous study [63], because the authors intended to compare the expressed genes in association with morphological and anatomical differences in FRUs and SRUs of *Gnetum* species. 

The total RNA for each sample was extracted using the RNeasyPlus Mini kit (Qiagen, Valencia, CA, USA). The concentration and integrity of extracted RNA were assessed using the NanoDrop 2000 (ThermoFisher Scientific, Shanghai, China) and Agilent Bio analyzer 2100 (Agilent Technologies, Beijing, China) systems, respectively. At least 1 μg of RNA for each sample was used, andRNA libraries wereestablished using the NEBNextUltraTM RNA Library Prep Kit (NEB, Ipswich, MA, USA) according to the manufacturer’s protocol. The samples were sequenced on an IlluminaHiSeq 2000 at Biomarker Technologies, Inc., Beijing, China. All transcript sequences have been deposited in the NCBI SRA database (reference number PRJNA539853).

### 2.6. Bioinformatics and Transcriptome Comparisons

The raw reads with poly-N and lows cores were deleted and the adaptors on each raw read were removed. All cleaned reads were mapped against the reference genome mentioned above using software HISAT2 v.2.1.0 [69] with the parameter of, at most, one nucleotide mismatch. The annotation and functional classification of identified genes were searched against the four databases (Swiss-Prot, Nr, GO and KEGG). The identified genes were further quantified with fragments-per-kilobase of transcript-per-million fragments mapped (FPKM). Prior to the differential expression analysis, the read counts of each RNA library were adjusted using the package edger [70], implemented in software R v.3.1.1 [71]. The differentially expressed genes (DEGs) were detected using the package EBSeq [72] with the threshold FDR<0.05 and |log_2_(FCvalues)| ≥ 1. The KEGG enrichment analyses of paired DEGs were performed using ggplot2, with the corrected *p* value < 0.05 as the threshold.

### 2.7. Integrated Proteome and Transcriptome Analyses

The integrated proteome and transcriptome analyses were performed using ggplot2. The integrated analyses were conducted based on the identical KO numbers shared by the DAPs between the female and male drops and the DEGs between the FRUs and SRUs. The ratios of paired DAPs and DEGs were represented in quadrants with four major groups: For Group I, the DAPs and DEGs had the same trend of changes (protein abundance and gene expression was simultaneously up- or down-regulated); for Group II, the DAPs and DEGs had opposing trends (the gene expression was up-regulated, while the protein abundance was down-regulated; or the gene expression was down-regulated, while the protein abundance was up-regulated); for Group III, the protein abundance was up/down-regulated, while the gene expression did not significantly change; for Group IV, the protein abundance did not significantly change, while the gene expression was up/down-regulated. The KEGG enrichment analyses of the paired DAPs and DEGs were performed using ggplot2, with the corrected *p* value < 0.05 as the threshold.

## 3. Results

### 3.1. Proteomic Diversity 

**Figure 2:**
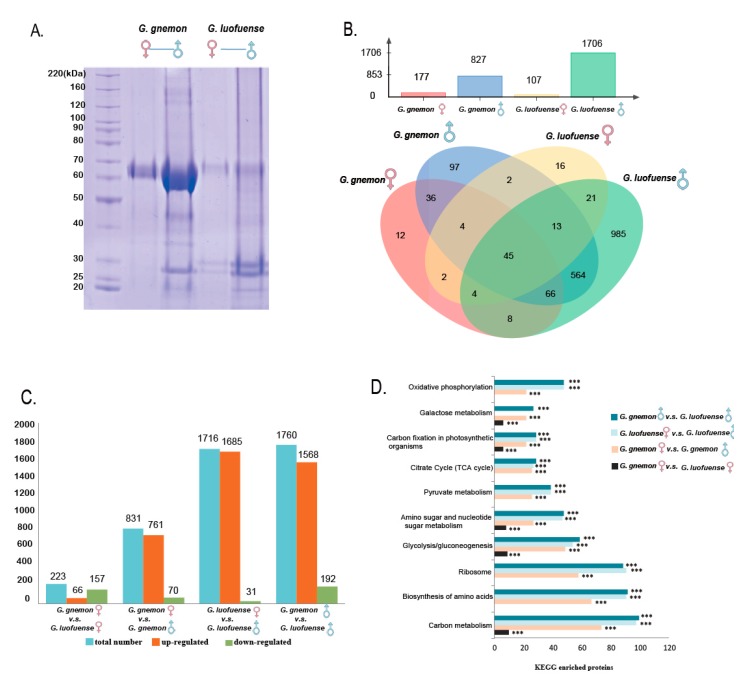
Proteomic data ofthe pollination drops of *Gnetum*. (**A**) Results of SDS-PAGE reveal the diversity and concentration of proteins in female and male drops of *G. gnemon* and *G. luofuense*. (**B**) Protein number detected in the female and male drops of *G. gnemon* and *G. luofuense* (above) and as a Venn graph (below), revealing the identical and differential proteins between the female and male drops of the two species. (**C**) Infra- and interspecific variation of differentially abundant proteins (DAPs) between the female and male drops of *G. gnemon* and *G. luofuense*. (**D**) These DAPs enriched in the top 10 Kyoto Encyclopedia of Genes and Genomes (KEGG) pathways; *** denotes corrected *p* values < 0.05.

The 1D SDS-PAGE results reveal the molecular weight of proteins accumulated to be 50–70 and 25–30 kDa in both the female and male drops of *G. gnemon* and *G. luofuense* (Figure 2A). It is noteworthy that the male drops of *G. gnemon* exhibit one pronounced protein band in the range of 25–30 kDa, whereas the equivalent drops in *G. luofuense* have two bands. Further, more bands were observed in the male drops of *G. gnemon* and *G. luofuense* than in comparable female drops. In addition, a total of 1875 proteins were identified from the four pollination drop samples, of which 52 were identified based on the newly detected genes from reproductive organ transcripts (Figure S1). The annotations and functional classification of all detected proteins identified using Swiss-Prot, Nr, GO and KEGG databases are listed in Table S1. The female and male drops in *G. gnemon* possessed 177 and 827 proteins, respectively, with the equivalent drops in *G. luofuense* having 107 and 1706 proteins (Figure 2B). The identities and differences of protein components among the four pollination drop samples are displayed in Figure 2B as a Venn diagram. The results show that 45 proteins were omnipresent, 12 and 97 proteins were unique to the female and male drops of *G. gnemon*, respectively, and 16 and 985 proteins were unique to the female and male drops of *G. luofuense*, respectively. 

### 3.2. Differentially Abundant Proteinsand KEGG Enrichment Analyses

The number of differentially abundant proteins (DAPs) common to female and male drops of *G. gnemon* was 831, with 761 up-regulated and 70 down-regulated (Figure 2C). In contrast, the number of DAPs shared between the female and male drops of *G. luofuense* were significantly larger (1716 proteins), with 1685 up-regulated and 31 down-regulated. The DAPs between the female and male drops of *G. gnemon*/*G. luofuense* were enriched in different KEGG pathways (Figure 2D, Supplementary Data Figure S1), namely, carbon metabolism (ID: ko01200, 74–98 proteins), the biosynthesis of amino acids (ID: ko01230, 67–91 proteins), ribosome (ID: ko03010, 58–91 proteins), glycolysis/gluconeogenesis (ID: ko00010, 49–54 proteins), and amino sugar and nucleotide sugar metabolism (ID: ko00520, 27–47 proteins). 

In addition, 223 DAPs were shared between the female drops of *G. gnemon* and *G. luofuense*, with 66 up-regulated and 157 down-regulated (Figure 2C). These DAPs were strictly enriched in several KEGG pathways with a few proteins (Figure 2D, Supplementary Data Figure S1), namely, carbon metabolism (10 proteins), glycolysis/gluconeogenesis (10 proteins) and amino sugar and nucleotide sugar metabolism (eight proteins). In contrast, significantly more DAPs (1760 proteins) were shared between the male drops of *G. gnemon* and *G. luofuense*, with 1568 up-regulated and 192 down-regulated. These DAPs were broadly enriched in different KEGG pathways with abundant proteins, namely, carbon metabolism (100 proteins), biosynthesis of amino acids (92 proteins), ribosome (89 proteins), glycolysis/gluconeogenesis (59 proteins) and amino sugar and nucleotidesugar metabolism (48 proteins).

### 3.3. Transcripts of Reproductive Organs

Four FRU and SRU transcriptomes of *G. gnemon* and *G. luofuense* were sequenced, generating 27.77 Gb of clean data for four libraries with Q30 values in the range 93.95–94.50% (Supplementary Data Table S2). After removing low-quality reads and reads with adaptors, this study generated ~53M and ~45M for FRUs and SRUs of *G. gnemon*, respectively, with GC contents ranging from 47.48–47.59%; and ~45M and ~42M for FRUs and SRUs of *G. luofuense*, respectively, with GC contents ranging from 48.59–49.12%. The clean reads were then mapped onto the reference genome, yielding 19,662,103 (37.04%) and 16,126,079 (36.14%) uniquely mapped reads for the FRUs and SRUs of *G. gnemon*, respectively. In contrast, *G. luofuense* possessed significantly more mapped reads: 39,107,072 (86.39%) and 37,850,972 (88.79%) for FRUs and SRUs, respectively. 

**Figure 3:**
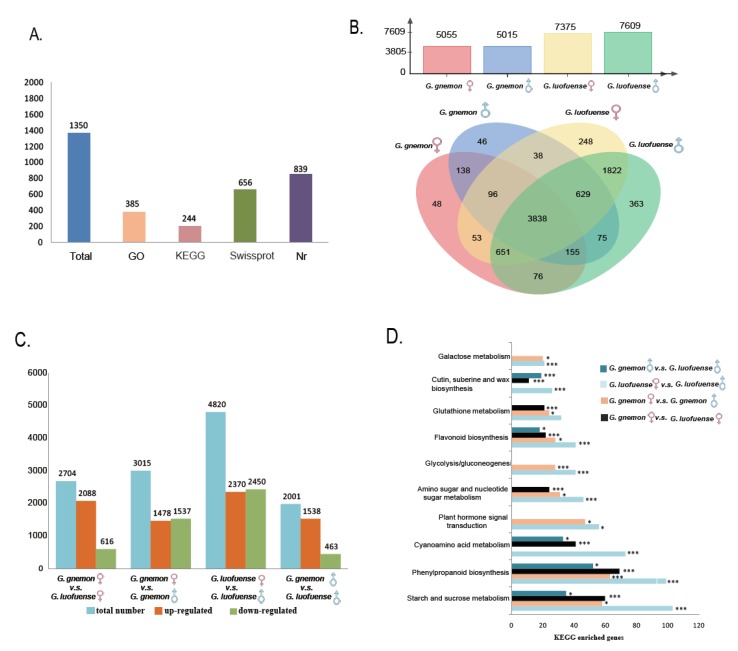
Transcriptomic data of reproductive organs in *Gnetum*. (**A**) Number of newly detected genes annotated against different databases. (**B**) Gene number detected in the fertile reproductive units (FRUs) and sterile reproductive units (SRUs) of *G. gnemon* and *G. luofuense* (above) and a Venn graph (below), revealing the identical and differential genes between the FRUs and SRUs of the two species. (**C**) Infra- and interspecific variation of differentially expressed genes (DEGs) between the FRUs and SRUs of *G. gnemon* and *G. luofuense*. (**D**) These DEGs enriched in the top 10 KEGG pathways; * represents *p* values < 0.05, ***denotes corrected *p* values < 0.05.

The RNA-seq data from the four libraries yielded in total 8276 genes, of which 1350 were newly detected (Figure 3A). The annotations and classification of all detected genes identified using Swiss-Prot, Nr, GO and KEGG databases are listed in Supplementary Data Table S3. In *G. gnemon*, 5055 and 5015 genes from the FRU and SRU transcripts were found, respectively, whereas, 7375 and 7609 genes were identified from the FRU and SRU transcripts in *G. luofuense* (Figure 3B). The identities and differences of the expressed genes in the four transcripts were represented asa Venn diagram (Figure 3B): The results show that 3838 genes were omnipresent, 48 and 46 genes were uniquely expressed in the FRUs and SRUs of *G. gnemon*, respectively, and 248 and 363 genes were unique to the FRUs and SRUs of *G. luofuense*, respectively.

### 3.4. Differentially Expressed Genes and KEGG Enrichment Analyses

A total of 3015 differentially expressed genes (DEGs) were identified between the FRUs and SRUs of *G. gnemon*, with 1478 up-regulated and 1531 down-regulated (Figure 3C). Considerably more DEGs were identified between the FRUs and SRUs of *G. luofuense* (4820 genes), with 2370 up-regulated and 2450 down-regulated. The DEGs between the FRUs and SRUs of *G. gnemon*/*G. luofuense* were enriched in different KEGG pathways (Figure 3D, Supplementary Data Figure S2), namely, starch and sucrose metabolism (ID: ko00500, 50–103 genes), phenylpropanoid biosynthesis (ID: ko00940, 63–99 genes), cyanoamino acid metabolism (ID: ko00460, 73 genes), plant hormone signal transduction (ID: ko04075, 47–56 genes) and amino sugar and nucleotide sugar metabolism (31–46 genes). 

In addition, the number of DEGs between the FRUs of *G. gnemon* and *G. luofuense* was 2704, with 2088 up-regulated and 616 down-regulated (Figure 3C). The DEGs were enriched in the KEGG pathways (Figure 3D, Figure S2), namely, starch and sucrose metabolism (60 genes), and phenylpropanoid biosynthesis (69 genes) and cyanoamino acid metabolism (41 genes). Moreover, 2001 DEGs were identified between the SRUs of *G. gnemon* and *G. luofuense*, with 1538 up-regulatedand 463 down-regulated. The DEGs were enriched in the following KEGG pathways: starch and sucrose metabolism (35 genes), phenylpropanoid biosynthesis (52 genes), cyanoamino acid metabolism (33 genes), flavonoid biosynthesis (ID: ko00941, 18 genes), and cutin, suberin and wax biosynthesis (ID: ko00073, 19 genes).

### 3.5. Integrated Proteome and Transcriptome Analyses

**Figure 4:**
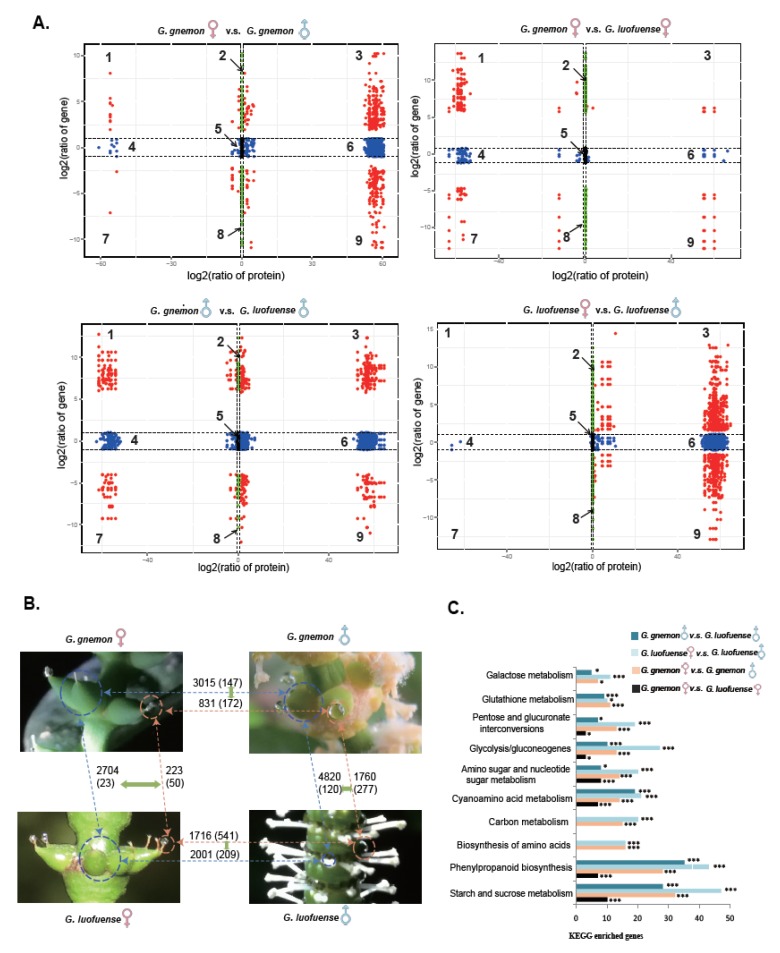
The results of integrated proteome and transcriptome analyses. (**A**) Quadrant graphs showing associations between the number of differentially abundant proteins (DAPs) in the pollination drops (x-axis, log^2^ ratio) and differentially expressed genes (DEGs) in the reproductive organs (y-axis, log^2^ ratio) of *G. gnemon* and *G. luofuense*. (**B**) Schematic graph revealing the paired DAPs (orange) and DEGs (blue) that have the same expression trend in the pollination drops and reproductive organs of *G. gnemon* and *G. luofuense* (highlighted with green arrows). (**C**) Paired DEGs enriched in the top 10 KEGG pathways; * represents *p* values < 0.05, *** denotes corrected *p* values < 0.05.

Four quadrants were applied to detect paired DAPs and DEGs that had the same trend of changes (Group I, as discussed in the Material and Methods; see zones 3 and 7” in the quadrants: Figure 4A). The results show that 172 DAPs between the female and male drops had the same change trend, with 147 DEGs between the FRUs and SRUs of *G. gnemon* (Figure 4B). Similarly, 541 DAPs had the same change trend between the female and male drops, with 209 DEGs between the FRUs and SRUs of *G. luofuense.* The paired DAPs and DEGs were enriched in the following KEGG gene pathways (Figure 4C, Figure S3): starch and sucrose metabolism (32–47 genes), phenylpropanoid biosynthesis (28–43 genes), biosynthesis of amino acids (16 proteins), carbon metabolism (15–20 proteins), and cyanoamino acid metabolism (14–21 genes).

The results also show that 50 DAPs shared between the female drops of *G. gnemon* and *G. luofuense* had a similar change trend, with 23 DEGs between the FRUs of the two species (Figure 4B). The paired DAPs and DEGs were enriched in the following KEGG gene pathways (Figure 4C, Figure S3): starch and sucrose metabolism (ten genes), phenylpropanoid biosynthesis (seven genes), and cyanoamino acid metabolism (seven genes). Moreover, 277 DAPs were found between the male drops of *G. gnemon,* and *G. luofuense* had an identical change trend, with 120 DEGs shared between the SRUs of the two species. The paired DAPs and DEGs were enriched in the following KEGG genes pathways: starch and sucrose metabolism (28 genes), phenylpropanoid biosynthesis (35 genes), and cyanoamino acid metabolism (19 genes). 

## 4. Discussion

### 4.1. Proteomic Diversity in the Pollination Drops of Gnetum

Our results highlight the strikingly diverse proteome in the pollination drops of *Gnetum* (Table 1). A previous study has reported that protein numbers in the female drops of *Ephedra* ranged from 6–20, depending on species [34]. The protein profile in the male drop of *E. foeminea*—the only species that possesses a morphologically bisexual structure—remains unknown. Furthermore, only one protein was detected in the female drops of *W. mirabilis* but the results—chitinase [31] and HOPZ-activated resistance enzyme [35]—differed in previous studies. In *Gnetum*, 17 and 25 proteins were previously identified in the female and male drops of *G. gnemon*, respectively [35]. In the present study, however, a total of 1875 proteins were identified from the female and male drops of *G. gnemon* and *G. luofuense*, with 177 and 107 proteins in the female drops and 827 and 1706 proteins in the male drops of the two species, respectively (Figure 2B). 

The considerable increase in protein numbers detected in the present study is partially ascribed to the application of recent label-free quantitative techniques. The method involves the direct digestion of pollination drop samples by trypsin rather than isotope labelling [73]. This is appropriate for the Gnetales which have a low concentration and diversity of proteins in their pollination drops [31,34]. The nuclear genome of *Gnetum* was furthermore taken into account during the process of protein identification. This has never been considered in previous studies. The advantage of applying a reference genome is the prevention of proteomic contamination from external pathogens and fungi within the pollination drops of *Gnetum* species. Comparison with a reference genome furthermore overcomes the problem of detected proteins having poor BLASTp matches or lacking matching genes in non-model gymnosperms [35]. In our case, approximately 120 detected proteins (accounting for 6% of the total) failed to be annotated, remaining to be addressed in the future when the proteomic database of gymnosperms is updated. The enriched knowledge of protein profiles in pollination drops therefore enables a better understanding of the pollination biology and reproductive evolution of *Gnetum*.

### 4.2. Infraspecific Variation of Protein Profiles

*Carbohydrate metabolism*. The pollination drops of the Gnetales are sugar-rich, with a higher total sugar concentration (TSC) than other gymnosperms [27]. The sugars in *G. gnemon* pollination drops are predominantly fructose (~78%), with less sucrose (~17%) and glucose (~5%).This provides a striking contrast with angiosperm nectar, which is typically sucrose dominated [27]. Moreover, it has been reported that carbohydrates, such as sucrose, are essential for mediating the osmolarity that affects pollen germination and pollen tube growth [74]. Our results show that the proteins in the pollination drops of *G. gnemon* and *G. luofuense* were more abundant in the male than the female drops (Figure 2B). The DAPs are involved in a series of carbohydrate metabolisms, such as carbon metabolism, glycolysis/gluconeogenesis, carbon fixation in photosynthetic organisms and galactosemetabolism (Figure 2D). In the male drops of *G. gnemon* and *G. luofuense*, for example, this studyfound glucose-6-phosphate isomerases that transform alpha-D-glucose to beta-d-fructose [75] and beta-fructofuranosidases and fructan 6-exohydrolases that degrade sucrose to beta-d-fructose and alpha-d-glucose [76,77]. Probably because of the effects of the carbohydrate proteins, the percentages of sugar types (sucrose, fructose and glucose) remain almost equal between the female and male drops of *G. gnemon* [27]. Despite the TSC in the male drops of *G. gnemon* being significantly lower than that in the female drops [27], the female and male drops produced by ovulate and staminate *Gnetum* plants might provide identical sugar profiles for attracting pollinators [27,35].

*Defense-related mechanisms*. As with other gymnosperms, the FRUs and SRUs of *Gnetum* are exposed, lacking the physical protection endowed by the carpel in angiosperms. The FRUs, which bear and develop young seeds accordingly, have to defend against external fungi and pathogens that may be introduced into the micropylar tube when the pollination drops are withdrawn. Previous studies report that defense-related proteins, a component of the secretome, are widespread in female drops of various gymnosperms. For example, thaumatin-like proteins are able to interrupt fungal cell wall formation [32,78] and hence protect the FRUs of conifers [31,32] and the Gnetales (e.g., *Ephedra minuta* Florin and *G. gnemon* [34,35]). Chitinases were reported in the female drops of *Welwitschia mirabilis* [31] and are involved in protecting the ovule against *Aspergillusniger* var. *phoenicis* [79]. Xylosidase and beta-glucodiase have moreover been reported to protect the ovules of conifers and *G. gnemon* [30,31,33,35]. The present study detected four defense-related proteins in the female drops of *G. gnemon* and *G. luofuense* (Table 1), indicating that pollination drops likely play an important role in FRU protection in *Gnetum*. Surprisingly, the four defense-related proteins were also detected in the male drops of *G. gnemon* and *G. luofuense* with up-regulated protein abundance (Table 1). Although the SRUs are not involved in seed production, the increased diversity and abundance of defense-related proteins in the male drops might play a role in protecting microsporangia or the entire male strobili. Furthermore, the defense-relate proteins in the male drops of *Gnetum* might promote sugar-rich but fungal/pathogen-free pollinator rewards.

*Amino acid metabolism.* In comparison with their anemophilous relatives, entomophilous and ambophilous gymnosperms typically possess a lower total amino acid content similar to that observed in angiosperm nectar [25,27,28]. The amino acid concentration is a major contributory factor affecting an insects’ taste sensation of sugar solutions, with high amino acid concentrations rendering the solution unpalatable for insects [80,81]. Some non-protein amino acids, such as beta-alanine, are nevertheless able to stimulate the neurophysiological system of insects and hence reinforce pollinator attraction [27,28]. Another amino acid, proline, triggers the salt cells in the insect’s labellar sensitive receptor and increases the frequency of pollinator visits [25,82]. Proline, moreover, provides a nutritional reward to insects, assisting with initial flight [83]. Our results support the hypothesis that the DAPs between the female and male drops of *G. gnemon/G. luofuense* are involved in the biosynthesis of amino acids (Figure 2D), with two proteins up-regulated: serine hydroxymethyltransferase, which catalyzes the reversible transformation from L-serine to glycine [84]; and pyrroline-5-carboxylate reductase, which reduces pyrroline-5-carboxylate to proline [85]. The absolute concentrations of alanine and proline in the male drops of *G. gnemon* were significantly higher than in the female drops [27], which are conjectured to function in pollinator attraction.

*Intracellular proteins*. Previous studies have shown that diverse intracellular proteins (degradome proteins) are widespread in the female drops of gymnosperms, including *Cephalotaxus* Sieb. et Zucc. ex Endl. [63], cycads and *Ginkgo* L. [35]. In the Gnetales, degradome proteins, such as ubiquitins, cyclophilin A, calmodulin, GTP-binding nuclear protein and elongation factors, have been reported in the female drops of *Ephedra* [34]. Several degradome proteins, such as ATPase subunit 1, ATPase and GTP-binding elongation factor Tu family protein have furthermore been documented in the male drops, but not the female drops, of *G. gnemon* [35]. Similarly, over 80% of annotated proteins detected in the male drops of *W. mirabilis* are intracellular, with none found in the female drops [35]. The present study found degradome proteins, such as elongation factors, expansions, histones and ribosomal proteins, present in both the female and male drops of *G. gnemon* and *G. luofuense*, whereas other degradome proteins, such as ubiquitins, calmodulin, GTP-binding nuclear protein and heat shock proteins, were uniquely expressed in the male drops (Table 1). Degradome proteins constitute most of the proteins detected in the male drops of both *G. gnemon* and *G. luofuense.* Since the process of drop exudation is accompanied by the formation of a shallow pollen chamber [39], the degradome proteins in the female and male drops of *Gnetum* are most likely derived from cellular relics as the degeneration of nucellus tissues, as in other gymnosperms [35]. Although the roles of degradome proteins in pollination biology are less well known compared to secretome proteins, they are nevertheless believed to be functionally significant. The accumulation of diverse degradome proteins in the male drops of *Gnetum* might act as a nutritive reward for pollinators. This study furthermore predicts that degradome proteins might interact with secretome proteins, resulting in a complex protein-protein network. A confirmation of the function, however, requires additional immunohistochemical studies. 

### 4.3. Interspecific Variation of Protein Profiles

Further, 223 DAPs were detected between the female drops of *G. gnemon* and *G. luofuense*, with several annotated proteins associated with carbohydrate metabolism in the KEGG enrichment analysis, namely, carbon metabolism, glycolysis/gluconeogenesis, amino sugar and nucleotide sugar metabolism, and galactose metabolism (Figure 2D). This is probably because sugar concentrations vary between arborescent and lianoid species: 3–13% in *G. gnemon*, versus 14.7% in *G. cuspidatum*, with the former suggested to be adapted to moth pollination [14]. In contrast, there are 1760 DAPs between the male drops of *G. gnemon* and *G. luofuense*, including the enriched KEGG carbohydrate metabolism pathways alluded to above, but also in amino acid synthesis, protein production (ribosome) and pyruvate metabolism (Figure 2D). It is evident that male drops are not as highly conserved as female drops in the Gnetales since the male drops are not responsible for pollen germination or pollen tube development [27,35]. The DAPs between the male drops of *G. gnemon* and *G. luofuense* might represent adaptations to different pollination systems: *G. luofuense* might favour diverse pollinators, such as moths, flies, beetles and cockroaches [45], whilst the relatively simple protein profiles in the male drops of *G. gnemon* might represent adaptation specifically to moths [14], although this requires testing.

### 4.4. Transcriptome Data and Newly Detected Genes

This is the first study to compare the transcriptomes of the FRUs and SRUs in the Gnetales. This study detected 1350 new genes, of which 52 aid protein detection (Figure 3A, Table S1). The newly detected genes assist in identifying new proteins in the pollination drops and facilitating transcriptome comparisons. Further, 5055 and 7375 genes were found from the FRUs, and 5015 and 7609 genes from the SRUs of *G. gnemon* and *G. luofuense*, respectively. The detection of fewer genes from *G. gnemon* is probably due to the low ratio of raw-read mapping against the reference genome (ranging from 37.38–38.44%: Table S2). *Gnetum gnemon* and the genome-reference species, *G. luofuense* (=*G. montanum*) [66], are phylogenetically distant, with the divergence time between Asian arborescent species and lianoid species estimated at c. 65 Ma (95% HPD, 81–48 Ma: [41]). There is accordingly a risk of overestimating DEGs between the FRUs/SRUs of *G. gnemon* and *G. luofuense*. 

### 4.5. Infraspecific Variation of Transcriptome Data

*Pollination drop formation*. Our results reveal that 16–18 of the DEGs between the FRUs and SRUs of *G. gnemon* and *G. luofuense* were annotated as starch and sucrose metabolism, of which alpha-amylases, sucrose synthases, glycosyl hydrolases and glycosyltransferase constituted the majority (Figure 3D, Supplementary Data Table S1). Starch was reported to accumulate in the nucellar cells of some gymnosperms prior to the production of pollination drops in *W. mirabilis* [86]. Alpha-amylases, which degrade starch, are present in the female drops of *Cephalotaxus* and *Ginkgo* [35,63]. The female drops of *Gnetum*, including *G. gnemon*, with a high TSC, are significantly larger than male drops with a low TSC [14,27]. The female drops of both specieswere furthermore observed to withdraw into a micropylar tube in the early morning, whereas the male drops gradually diminished, probably due to evaporation. The contrasting manner of pollination drop formation between the FRUs and SRUs of *Gnetum* is similar to that previously reported in *Welwitschia mirabilis* [12,35]. The different sizes and secretion manner of the female and male drops of *Gnetum* might be mediated by different osmotic potential due to different sugar concentrations. 

*MADS-box genes*. Regardless of species, the FRUs and SRUs are structurally different in *Gnetum*, with the former possessing three envelopes, whereas the latter has only two [19,55,56,57]. It is known that MADS-box genes are important for determining sexual identity and the development of reproductive organs of *Gnetum* [60,61,62,87]. MADS-box genes consist of two types—Type I and Type II—which comprise MIKC* and MIKC^c^ group genes [88,89]. Type II MIKC^c^ group genes perform varied roles in manipulating the reproductive development of *Gnetum* [60,87,90]. It was found that the *AG*-like gene *GGM3* was strongly expressed in both the FRUs and SRUs of *G. gnemon* and *G. luofuense*, consistent with the previous study [87]. It was further found that the *AGL17*-like gene *GGM6* was weakly expressed in the FRUs but strongly expressed in the SRUs of *G. gnemon* and *G. luofuense*. This was again consistent with previous studies [60,87,90]. Two *DEF/GLO*-like genes, *GGM2* and *GGM15*, were furthermore strictly expressed in the SRUs of *G. gnemon* and *G. luofuense*, as in previous studies [60,90]. 

### 4.6. Interspecific Variation Intranscriptome Data

The differences in the gross morphology of the SRUs of *G. gnemon* and *G. luofuense* are very pronounced: The former is exposed and funnel-shaped, whilst the latter is hidden within an involucral collar and is obliquely and/or narrowly ovoid, resembling the SRUs of *G. cuspidatum* and *G. parvifoium* [19,46,47]. This study detected 2001 DEGs between the SRUs of *G. gnemon* and *G. luofuense* (Figure 3C), of which 52 were annotated in the KEGG pathways (phenylpropanoid biosynthesis; Figure 3D). The seed envelopes of the FRUs and SRUs are both traversed by vascular bundles as described for several species of *Gnetum* [58,91,92]. The genes that encode phenylpropanoid biosynthesis, such as peroxidase, cause lignification of cell walls duringthe secondary thickening of xylem vessels [93]. Another gene that encodes cinnamyl alcohol dehydrogenase (CAD) is also responsible for xylem lignifications [94,95]. Moreover, 19 DEGs between the SRUs of *G. gnemon* and *G. luofuense* were enriched in cutin and suberinbiosynthesis (Figure 3D). Among the DEGs, peroxygenase was important in the biosynthesis of cutins that consist of multiple cuticles [96]. The DEGs detected were probably involved in the different gross morphologies between the SRUs of *G. gnemon* and *G. luofuense*.

### 4.7. Inferred Reproductive Evolution of Gnetum

Insect pollination has been a key driver of angiosperm diversification [97,98]. The antiquity of the Gnetales-insect pollination interaction is evidenced by its first fossil record in the Triassic and consequential increase in species diversity in the Early Cretaceous, with the entire process occurring synchronous with the radiation of angiosperms [99]. Since molecular phylogenies are unambiguous for indicating a sister-group relationship between the Gnetales and conifers [41,100,101,102], entomophily probably evolved in *Gnetum* in parallel with angiosperms. In the context of gnetalean phylogeny, entomophily is likely to be ancestral, with anemophily the derived condition [9,11,19]. Amongst the extant Gnetales, the insect pollination syndrome shared by *E. foeminea*, *W. mirabilis* and the majority of *Gnetum* species is characterized by the presence/absence of male drops [19]. 

Our results reveal that 172 DAPs had the same expression trend, with 147 DEGs between the FRUs and SRUs within *G. gnemon*, and with corresponding data for 541 DAPs and 209 DEGs for *G. luofuense* (Figure 4B). These paired DAPs and DEGs were enriched in the KEGG pathways, namely, starch and sucrose metabolism, glycolysis/gluconeogenesis, amino acid biosynthesis and phenylpropanoid biosynthesis (Figure 3B). The results suggest that insect pollination might impose different selection on the female/male drops and FRUs and SRUs within specific *Gnetum* species, although this statement requires further empirical pollination ecological studies. In addition, our results show that 50 DAPs between the female drops of *G. gnemon* and *G. luofuense* and 23 DEGs between the FRUs of the two species reveal the same expression trend (Figure 4B). There were few paired DAPs and DEGs and these were strictly enriched in the KEGG pathways, namely, starch and sucrose metabolism, phenylpropanoid biosynthesis, glycolysis/gluconeogenes, and amino sugar and nucleotide sugar metabolism (Figure 4C). However, 277 DAPs were found between the male drops of *G. gnemon*, and *G. luofuense* had the same trend with 120 DEGs between the SRUs of the two species (Figure 4B). These paired DAPs and DEGs were broadly enriched in the KEGG pathways, including starch and sucrose metabolism, phenylpropanoid biosynthesis, amino sugar and nucleotide sugar metabolism, and cyanoamino acid metabolism (Figure 4C).The divergence between arborescent and lianoid *Gnetum* species becomes more pronounced because the SRUs and male drops are probably more sensitive to insect pollination. The biochemistry of gnetalean pollination drops appears to have an impact on the selection exerted by insects, with high sugar concentrations, low amino acid composition, and the presence of typical sugar-amino acid profiles [27], but this requires testing in future studies.

## 5. Conclusions

This is the first study to investigate the underlying molecular mechanisms that drive insect-pollinated adaptations in gymnosperms. A recently developed methodology, label-free quantitative proteomics enables the more diverse protein profiles in the female and male drops of *G. gnemon* and *G. luofuense* to be revealed. Our results corroborate previous research that has indicated that male drops have more abundant proteins than female drops in the two species, probably due to entomophilous adaptations. The interspecific variation of the protein profiles of the female/male drops is furthermore likely to be a response to the different pollination syndromes of *G. gnemon* and *G. luofuense*. In addition, the transcriptome comparisons of the FRUs and SRUs within species exhibit striking levels of variation, with the differentially expressed genes likely to be involved in the development of reproductive organs and the formation of pollination drops. The interspecific variation of the expressed genes occurs between the SRUs of *G. gnemon* and *G. luofuense*, probably reflecting morphological and anatomical differences. The integrated protein-transcript analyses reveal that the female drops and the FRUs are relatively conserved compared to the male drops and SRUs, probably because the latter have been evolutionarily more responsive to insect pollination.

## Figures and Tables

**Table 1:** Thirtycommonly seen proteins identified in the pollination drops of gymnosperms.

	Groups	Cycads			Ginkgo	Conifers							Gnetales														
Proteins		CH	ZF	CR	GB	CS	CK	CL	JC	JO	PM	TM	ED	EF	EM	ET	EL	EM	EP	WM(F)	WM(M)	GG(F)	GG(M)	GG(F)*	GG(M)*	GL(F)*	GL(M)*
Alpha(beta)-galactosidase		X	X		X	X	X				X				X	X			X			X		X	X	X	X
Alpha-amylase					X	X	X										X		X						X		X
Alpha-fucosidase																						X		X	X	X	X
Arabidongalactan protein		X			X							X													X	X	X
Aspartyl protease		X	X		X						X					X	X		X			X		X	X	X	X
ATPases																					X		X		X		X
Beta-glucanse (or endoglucanase)																						X		X	X	X	X
Beta-glucosidase		X			X		X	X	X							X		X						X	X	X	X
Beta-hexosaminidase			X		X																			X	X	X	X
Calmodulin						X							X						X						X		X
Chitinase										X				X		X				X		X		X	X	X	X
Cystatin																						X		X	X	X	X
Cysteine protease		X	X		X																		X	X	X		X
Dehydrogenases						X	X									X					X				X		X
Elongation factors			X		X	X							X	X		X					X			X	X		X
Expansin					X																			X	X	X	X
Fasciclin-like arabinogalactan		X			X																				X	X	X
Glycosylhydrolase		X	X	X	X									X		X						X		X	X		
GTP-binding nuclear protein													X					X							X		X
Heat shock protein					X	X							X								X				X		X
Histones						X							X	X		X								X	X		X
Invertase											X																X
Peroxidase		X		X		X	X				X					X	X		X		X	X			X		X
Polygalacturonas(-like) protein							X																	X	X	X	X
Ribosomal proteins					X																X			X	X	X	X
Serine carboxypeptidase (-like) protein		X									X			X		X		X					X	X	X	X	X
Subtilisin-like proteinase (serine endopeptidase)		X	X					X	X													X	X	X	X	X	X
Thaumatin-like protein		X	X		X	X	X	X	X	X		X			X							X		X	X	X	X
Ubiquitins				X	X																				X		X
Xylosidase											X			X	X	X			X					X	X	X	X

Note: 1. CH-female drops of *Ceratozamia hildae* G.P.Landry & M.C. Wilson, ZF-female drops of *Zamia furfuracea* L.f. ex Aiton, CR-female drops of *Cycas rumphii* Miq., GB-female drops of *Ginkgo biloba* L., WM(M)-male drops of *Welwitschia mirabilis* Hook.f., GG(F)-female drops of *Gnetum gnemon* L., GG(M)-male drops of *G. gnemon*, data were published in Prior et al. 2018; 2. CS-female drops of *Cephalotaxus sinensis* (Rehder & E.H.Wilson) H.L.Li, CK-female drops of *C. koreana* Nakai, data were published in Pirone-Davies et al. 2016; 3. CL-female drops of *Chamaecyparis lawsoniana* (A.Murraybis) Parl., JC-female drops of *Juniperus communis* L., JO-female drops of *J. oxycedrus* L., WM(F)-female drops of *W. mirabilis* (F), data were published in Wagner et al. 2007; 4. PM-female drops of *Pseudotsuga menziesii* (Mirb.) Franco, data were published in Poulis et al. 2005; 5. TM-female drops of *Taxus*×*media*, O’Leary et al. 2007; 6. ED-female drops of *Ephedra distachya* L., EF-female drops of *E. foeminea* Forssk., EM-female drops of *E. minuta* Florin, ET-female drops of *E. trifurca* Torr. ex S.Watson, EL-female drops of *E. likiangensis* Florin, EM-female drops of *E. monosperma* J.G.Gmel. ex C.A.Mey., EP-female drops of *E. compacta* Rose, data were published in von-Aderkas et al. 2015; 7. GG(F)*-female drops of *G. gnemon*, GG(M)*-male drops of *G. gnemon*, GP(F)*-female drops of *G. luofuense*, GL(M)*-male drops of *G. luofuense*. The results derived from the present study.

## Supplementary Materials

The following are available online at https://www.mdpi.com/2073-4425/10/10/800/s1, Figure S1: KEGG enrichment analyses of the differentially abundant proteins (DAPs) between the female and male drops of *G.* spp. and between the female/drops of different species; Figure S2: KEGG enrichment analyses of the differentially expressed genes (DEGs) between the fertile and sterile ovules of *G.* spp. and between the female/drops of different species; Figure S3: KEGG enrichment analyses of differentially expressed genes (DEGs) that have the same trend of changes with the differentially abundant proteins (DAPs); Table S1: Protein types and amount identified in the pollination drops of *G.* spp. and their annotations using Label-free technique; Table S2: Information about transcriptome assembly and genome mapping; Table S3, Detected genes from reproductive transcripts of *G.* spp. and their annotations.
