# Supplementary material for: Pollination Drop Proteome and Reproductive Organ Transcriptome Comparison in Gnetum Reveals Entomophilous Adaptation
Source: Genes (Basel). 2019 Oct 12;10(10):800. doi: 10.3390/genes10100800 (PMC6826882; doi:10.3390/genes10100800)
Supplement: Supplementary file 1 [file genes-10-00800-s001.zip › Fig. S2 Hou et al.doc]

**Figure S2. KEGG enrichment analyses of the differentially expressed genes (DEGs) between the fertile and sterile ovules of *G. spp* and between the female/drops of different species**

1. **FRUs of *G. gnemon* v.s. SRUs of *G. gnemon***

2. **FRUs of *G. gnemon* v.s. FRUs of *G. luofuense***

3. **SRUs of *G. gnemon* v.s. SRUs of *G. luofuense***

4. **FRUs of *G. luofuense* v.s. SRUs of *G. luofuense***
