# Supplementary material for: Pollination Drop Proteome and Reproductive Organ Transcriptome Comparison in Gnetum Reveals Entomophilous Adaptation
Source: Genes (Basel). 2019 Oct 12;10(10):800. doi: 10.3390/genes10100800 (PMC6826882; doi:10.3390/genes10100800)
Supplement: Supplementary file 1 [file genes-10-00800-s001.zip › Table S2 Hou et al.doc]

**Table S2. Information about transcriptome assembly and genome mapping**

| **Transcriptome samples** | **Clean reads** | **GC content** | **%≥Q30** | **Mapped reads**  **(ratio%)** | **Unique mapped reads** |
| --- | --- | --- | --- | --- | --- |
| **FRUs of *G. gnemon*** | 53,076,334 | 47.48% | 93.95% | 20,405,134  (38.44%) | 19,662,103  (37.04%) |
| **SRUs of *G. gnemon*** | 44,620,024 | 47.59% | 94.04% | 16,678,872  (37.38%) | 16,126,079  (36.14%) |
| **FRUs of *G. luofuense*** | 45,265,928 | 49.12% | 94.50% | 40,401,860  (89.25%) | 39,107,072  (86.39%) |
| **SRUs of *G. luofuense*** | 42,629,818 | 48.59% | 92.86% | 38,920,613  (91.30%) | 37,850,972  (88.79%) |
